# Integrating child health services into malaria control services of village malaria workers in remote Cambodia: service utilization and knowledge of malaria management of caregivers

**DOI:** 10.1186/1475-2875-12-292

**Published:** 2013-08-23

**Authors:** Aya Hasegawa, Junko Yasuoka, Po Ly, Chea Nguon, Masamine Jimba

**Affiliations:** 1 Department of Community and Global Health, Graduate School of Medicine, The University of Tokyo, 7-3-1 Hongo, 113-0033 Bunkyo-ku, Tokyo, Japan; 2 National Centre for Parasitology, Entomology and Malaria Control, 372 Monivong Boulevard, Phnom Penh, Cambodia

**Keywords:** Malaria, Child health, Communicable diseases, Quality of service, Health service utilization, Community health worker, Cambodia

## Abstract

**Background:**

Malaria and other communicable diseases remain major threats in developing countries. In Cambodia, village malaria workers (VMWs) have been providing malaria control services in remote villages to cope with the disease threats. In 2009, the VMW project integrated child health services into the original malaria control services. However, little has been studied about the utilization of VMWs’ child health services. This study aimed to identify determinants of caregivers’ VMW service utilization for childhood illness and caregivers’ knowledge of malaria management.

**Methods:**

A cross-sectional study was conducted in 36 VMW villages of Kampot and Kampong Thom provinces in July-September 2012. An equal number of VMW villages with malaria control services only (M) and those with malaria control plus child health services (M+C) were selected from each province. Using structured questionnaires, 800 caregivers of children under five and 36 VMWs, one of the two VMWs who was providing VMW services in each study village were interviewed.

**Results:**

Among the caregivers, 23% in M villages and 52% in M+C villages utilized VMW services for childhood illnesses. Determinants of caregivers’ utilization of VMWs in M villages included their VMWs’ length of experience (AOR = 11.80, 95% confidence interval [CI] = 4.46-31.19) and VMWs’ service quality (AOR = 2.04, CI = 1.01-4.11). In M+C villages, VMWs’ length of experience (AOR = 2.44, CI = 1.52-3.94) and caregivers’ wealth index (AOR = 0.35, CI = 0.18-0.68) were associated with VMW service utilization. Meanwhile, better service quality of VMWs (AOR = 3.21, CI = 1.34-7.66) and caregivers’ literacy (AOR = 9.91, CI = 4.66-21.05) were positively associated with caregivers’ knowledge of malaria management.

**Conclusions:**

VMWs’ service quality and length of experience are important determinants of caregivers’ utilization of VMWs’ child health services and their knowledge of malaria management. Caregivers are seeking VMWs’ support for childhood illnesses even if they are providing only malaria control services. This underlines the importance of scaling-up VMWs’ capacity by adding child health services of good quality, which will result in improving child health status in remote Cambodia.

## Background

Despite growing concern over chronic non-communicable diseases, malaria and other communicable diseases remain major threats in developing countries. In Cambodia, more than three million people were at risk of malaria transmission and approximately 50,000 malaria cases were confirmed in 2010; this is the second highest number of any country in the Western Pacific Region. Moreover, Cambodia is among those countries suffering from multidrug-resistant malaria arising through the proliferation of artemisinin-tolerant malaria parasites [1-3]. Adding to the burden, the high-risk areas for malaria transmission are concentrated in remote forested areas which are common place to live among the poor, ethnic minorities and migrants who have limited access to public health services [4,5].

Cambodia has made marked progress to decrease under-five mortality from 117 to 43 deaths per 1,000 live births in the past 20 years, but it is still high compared to the regional average of 29 deaths per 1,000 live births [6]. Acute respiratory infections (ARI), diarrhoeal diseases, and febrile illnesses including malaria contribute to 30, 27, and 11% of under-five deaths, respectively [3,7]. Sociodemographic factors, such as living in remote areas, households’ low level of wealth, and caregivers’ low education level, are known to contribute to poor child health [3,7,8]. It is thus critically important to deliver effective childhood illness management interventions to these vulnerable households to improve the chances of child survival [9-11].

Adding to the communicable disease threats and burden of high under-five mortality, Cambodia is one of the 57 countries identified as facing a health workforce crisis and has been suffering from a severe health workforce shortage [12]. The country has the greatest subnational inequities in the distribution of medical doctors among the ten countries of the Association of Southeast Asian Nations (ASEAN) [13]. Most public health facilities are understaffed or do not have enough health care providers with sufficient clinical skills to deliver the services necessary in remote areas [14,15]. Consequently, most vulnerable populations do not have access to appropriate health services by well-trained health professionals.

Coverage of evidence-based interventions against communicable diseases is still low in Cambodia, especially in remote areas [15]. In such areas, community health workers (CHWs) indeed play a vital role in connecting communities to health services that are available, accessible, appropriate, and of good quality [16]. CHWs can play crucial roles to address the health workforce shortages, especially in low- and middle-income countries [17-20]. Moreover, when CHWs can provide integrated interventions rather than vertical single-disease initiatives, the child health outcomes are generally better in hard-to-reach and resource-poor settings, where a large percentage of child deaths occur [19,21-25].

CHWs are commonly sought out as the first-line of care in remote areas, whereas private clinics and private pharmacies are more commonly accessed in urban areas in Cambodia [7]. Therefore, strengthening the capacity of CHWs to provide good quality of care and to deliver the essential interventions represents one of the key ways to improve the public health status of remote village residents.

The Cambodian Government’s National Centre for Parasitology, Entomology and Malaria Control (CNM) started a community-based malaria control project called the Village Malaria Worker (VMW) project as part of its National Malaria Control Programme in 2001 [4]. The VMWs are CHWs who were trained to effectively deliver malaria prevention and treatment services to remote villages. The VMW project was rolled out initially in 36 villages of a remote province, Rattanakiri. CNM staff members identified target VMW villages, each of which was located more than a 5-km or one-hour walk from the nearest public health centre. As part of the process, then and now, two villagers (one male and one female) are selected as VMWs in each village through community consensus. The VMWs are then trained in malaria control interventions including prevention, diagnosis, and treatment. The CNM staff members and district health officers supply essential items, such as rapid diagnostic tests (RDTs) and artemisinin combination therapy (ACT), and they supervise and monitor VMW activities monthly [4,26].

The VMW project has been gradually scaled up and now plays an important role in reducing malaria morbidity and mortality [3]. It was expanded to 315 villages in seven provinces in 2004, further extended to 1,394 villages in 2009, and finally reached 1,528 villages in 17 provinces in 2012. The VMW project has improved knowledge and practices of VMWs to prevent malaria and to provide early diagnosis and treatment in remote malaria endemic villages [16,27]. VMWs can now provide malaria control services comparable in quality to those of the local health facilities [28]. In March 2011, the government of Cambodia formally committed itself to eliminate malaria by the year 2025, identifying the VMW project as one of the key strategies to meet that goal [4].

The VMW project added child health services on top of their malaria control services starting in 2009 [3,8]. VMWs in 400 villages, who had been trained and started providing malaria control services by 2008, were given an additional training to provide child health services in 2009. This new approach was technically and financially supported by the World Health Organization (WHO) and the Global Fund for AIDS, TB and Malaria (GFATM) [4]. Since VMWs had already experienced success in delivering the essential interventions for malaria control in remote villages, they were then expected to deliver the essential interventions for childhood illnesses, too.

The additional roles of VMWs in implementing child health services are as follows: to manage childhood illnesses, to prescribe and provide basic medications (antibiotics; cotrimoxazole, antipyretics; paracetamol, oral rehydration salts (ORS) and zinc), to refer severe cases to the appropriate health centres, and to provide preventive education such as promotion of breastfeeding and sanitation skills. As a result, a total of 15,898 children received child health services from VMWs in 2011 [4,26].

At this stage, it is important to take stock and evaluate how the integration of malaria control services and child health services have influenced service providers and service users. VMWs in M villages only provide malaria control services, but villagers visited VMWs to seek care for childhood illnesses [4]. With respect to service providers, initial findings suggested that the integration has increased VMWs’ motivation and improved the quality of their malaria control services [26]. However, no study has yet been conducted to focus on the experience of service users after the introduction of the new child health services elements. Therefore, this study aimed to identify determinants of caregivers’ VMW service utilization for childhood illnesses. It also examined the association between VMWs’ service quality and caregivers’ knowledge of childhood malaria management.

## Methods

### Study area

A cross-sectional study was conducted in 36 villages of two remote malaria-endemic provinces in Cambodia: Kampot and Kampong Thom. These 36 villages were selected out of 212 villages included in the VMW project sites in these two provinces in Cambodia. Kampot is located about 150 km south and Kampong Thom about 160 km north of the capital city, Phnom Penh. Kampot and Kampong Thom are similar to each other in terms of several demographic and epidemiological factors, including population size (585,850 and 631,409) [29], under-five mortality rate (73 and 67 deaths per 1,000 births respectively, compared with the national average of 54 deaths) [7], and number of malaria positive cases per VMW village (2.7 and 2.6 cases per month respectively, compared with the average for all VMW villages of 2.5 cases monthly). In Kampot, there were 36 villages with malaria control services only (M villages) and 27 villages with both malaria control services and child health services (M+C villages), while in Kampong Thom, there were 137 M and 12 M+C villages.

From each province, 18 villages (nine M villages and nine M+C villages) were selected as study sites based on the distance from the nearest public health centres. To this end, a list of VMW villages in the selected two provinces was provided by CNM. The villages were then sorted by distance from the nearest public health centre. The nine farthest villages in each type of service group were selected from each province.

### Participants

This study had two categories of participants. Members of the first category were primary caregivers of children under five years of age exhibiting any symptoms of ARI, diarrhoeal diseases, or febrile illnesses within the three months prior to the survey. In cases where there was more than one eligible child in a household, the child who had experienced symptoms most recently was selected as the focus of the questions. Caregivers under the age of 18 were excluded.

The sample size of caregiver participants was calculated using Power and Precision software (Biostat, Englewood, New Jersey, USA), based on 40% VMW service utilization in M villages [28] and the assumption of an approximately 10% higher utilization in M+C villages, with alpha set at 0.05 and 80% power. This resulted in a minimum sample size of 388 caregivers to be surveyed per service type. In order to counteract the effects of missing data, 400 caregivers per service type (200 from each province) were included in this survey. The sample size of each village was calculated based on population proportions.

Members of the second category were one of the two VMWs who was leading the VMW service activities in each study village.

In total, 800 caregivers and 36 VMWs were recruited from 36 villages in two provinces.

### Data collection

Data were collected from July to September 2012. The data collection period coincided with Cambodia’s heavy rainy season, when malaria transmission reaches its peak, and ARI and diarrhoea incidences among children likewise swell.

One supervisor and seven interviewers were hired and trained to conduct face-to-face interviews. All of the interviewers were native speakers of the Khmer language, were fully conversant in and sensitive to the cultural context of the study site, and had experience in conducting malaria-related household surveys in remote villages.

To identify the determinants of the service utilization and caregivers’ knowledge of childhood malaria management among those who live geographically available distance from the VMW services, the starting point for sampling household in each village was set at the VMW’s residence. Seven interviewers started sampling from the nearest house from the VMW’s residence and visited the next closest house to the last house sampled until the target sample size of each village was reached.

### Measures

A total of 800 caregivers and 36 VMWs (one from each study village) were interviewed, using structured questionnaires. Both caregiver- and VMW-targeted questionnaires were prepared originally in English, translated into Khmer, and then back-translated into English by Cambodian public health and malaria experts to ensure content fidelity. A pre-test was conducted prior to the survey in a village near to and of comparable demographic composition to the study site.

Dependent variables were caregivers’ utilization of VMW services and caregivers’ knowledge of childhood malaria management. To measure caregivers’ utilization of VMW services, caregivers were first asked “Have any of your children under five had fever, diarrhoea or fast/difficult breathing in the last 3 months” and then asked “Have you taken your children to a VMW to seek treatment?”. The following symptoms defined as ARI related symptoms: cough, sore throat, runny nose, difficult breathing, and fast and short breathing [7]. Diarrhoeal diseases were defined as three or more semi-liquid stools or bloody semi-liquid stools over a 24-hour period [30]. Febrile illnesses included suspected cases of malaria were defined broadly as diseases associated with fever [7].

Caregivers’ knowledge of childhood malaria management was measured by the malaria knowledge index. This index was developed based on caregivers’ answers to survey questions regarding five aspects of malaria-related knowledge: prevention, cause, symptoms, diagnosis, and treatment [31-33]. One point was awarded for each correct answer, and knowledge of each aspect was assessed by a single item score Item scores were summed to yield the total score for the resulting five-item index, Cronbach’s alpha for which was 0.71. Scores on this knowledge index were divided into two categories, high and low, with the cutoff point set based on the median.

Independent variables included VMW’s service quality, which was measured by using a quality index [26,33] based on VMWs’ answers to survey questions. The quality index composed of five items: active detection, diagnosis and treatment, perception of anti-malarials, follow-up, and dissemination of preventive measures. Item scores were summed to create the five-item index, Cronbach’s alpha for which was 0.60. VMWs who had score equal to or higher than median were considered to be providing better quality of service in each M and M+C village.

Data on the sociodemographic characteristics of caregivers and their children were obtained using the household and women questionnaires of the Cambodia Demographic and Health Survey (CDHS) 2010 [7]. Caregivers were assigned to socio-economic status terciles based on household assets and housing characteristics determined by principal component analysis [34]. Caregivers’ care-seeking behaviour was measured using the CDHS and integrated management of childhood illness multi-country evaluation household survey questionnaire [7,32].

Data on VMWs’ characteristics and experience including age, sex, occupation, length of education, and working experiences were collected using a VMW survey questionnaire developed with reference to previous relevant studies [26,33]. Distance from the nearest health centre to each village was obtained from the official records collected by the VMW project.

### Data analysis

Data from 800 caregivers and 36 VMWs were used to examine the benefits of VMW services for childhood illness management in remote villages of Cambodia. As M and M+C villages varied in their characteristics, results for the two village types were analysed separately except for the items pertaining to common service elements.

Differences between caregivers who used VMW services and those who did not were analysed by T-tests and Chi-square tests. To identify the determinants of VMW service utilization, multiple logistic regression analysis was conducted, adjusting for potential confounders. Another multiple logistic regression analysis was conducted to identify the relationship between caregivers’ knowledge of childhood malaria management and VMW’s service quality.

All continuous variables were converted to dichotomous variables at the point of the sample median for multiple logistic regression analysis [35]. The Variance Inflation Coefficient and the Spearman rank order correlation were used to assess multicollinearity between potential confounders for regression analysis; a rho of 0.5 or higher was taken as an indication of multicollinearity [36]. Statistical significance was set at p-value less than 0.05. All statistical analyses were performed using PASW18 (SPSS Inc, Chicago, Illinois, USA).

### Ethical considerations

Ethical approval was obtained from the Research Ethics Committee of the Graduate School of Medicine, the University of Tokyo (the approval number: 3828). The study protocol, consent form, information sheet, and survey questionnaires were also reviewed by the CNM Institutional Review Board, National Ethics Committee for Health Research, Cambodia, which issued an exemption letter. All participants were provided with information regarding the study prior to the survey and participated voluntarily. Written informed consent was obtained and confidentiality assured for all the participants.

## Results

### Sociodemographic characteristics of participants

In total, 800 caregivers participated in this study (Table 1). All participants were of Khmer ethnicity and 91.6% of them were farmers. The majority of the primary caregivers of children were mothers (95.8%) and were also involved in the decision-making process to seek care for childhood illnesses (mother only or both parents involved in decision-making process: 96.2%). Ages of caregivers ranged from 18 to 79 (mean 29.8) years. Distance from households to the closest public health centre ranged from 5 to 77 (mean 27.0) km. The number of household in each village varied from 41 to 793 (mean 214). Accordingly, the number of surveyed household per village varied from 6 to 63 (mean 22). Among the surveyed households, 76% had one, 23% had two, and 1% had three or more children under five. Compared to caregivers from M villages, caregivers from M+C villages had larger numbers of children under five years at home (M 1.2 *vs* M+C 1.3, p = 0.001), lived farther from the closest public health centre (M 22.3 km *vs* M+C 31.7 km, p <0.001), had lower education levels (illiterate: M 39.8% *vs* M+C 58.8%, p <0.001), and belonged to the low wealth category (M 24.0% *vs* M+C 28.8%, p <0.001).

**Table 1:** Sociodemographic characteristics and care-seeking behavior of participants

**Variables**	**Total (n=800)**		**M (n=400)**		**M+C (n=400)**		
	**n/**$\left(\bar{X}\right)$	**%/(SD)**	**n/**$\left(\bar{X}\right)$	**%/(SD)**	**n/**$\left(\bar{X}\right)$	**%/(SD)**	**p-value**
Caregiver’s age (years) (Mean:$\bar{X}$, SD)	(29.8)	(7.8)	(29.9)	(8.1)	(29.7)	(7.5)	0.818
Number of children U5 (Mean:$\bar{X}$, SD)	(1.3)	(0.5)	(1.2)	(0.4)	(1.3)	(0.5)	0.001
Distance from health centre (km) (Mean:$\bar{X}$, SD)	(27.0)	(23.0)	(22.3)	(20.9)	(31.7)	(24.0)	<0.001
Caregiver’s literacy							
Illiterate	394	49.3	159	39.8	235	58.8	<0.001
Literate	406	50.8	241	60.3	165	41.3	
Caregiver’s wealth index							
Low	211	26.4	96	24.0	115	28.8	<0.001
Middle	340	42.5	149	37.3	191	47.8	
High	249	31.1	155	38.8	94	23.5	
VMW’s experience (months) (Mean:$\bar{X}$, SD)	(60.7)	(28.5)	(37.9)	(5.5)	(83.5)	(23.4)	<0.001
Know VMW in village							
Yes	754	94.3	360	90.0	394	98.5	<0.001
Ever used VMW services							
Yes	599	74.9	276	69.0	323	80.8	<0.001
Used VMW for childhood illnesses*							
Yes	298	37.3	91	22.8	207	51.8	<0.001
Child age (months) (Mean:$\bar{X}$, SD)	(27.8)	(16.0)	(29)	(15.9)	(27)	(16.0)	0.083
Most recent symptoms							
ARI related symptoms	696	87.0	361	90.3	335	83.8	0.006
Diarrhoea	238	29.8	93	23.3	145	36.3	<0.001
Fever/Malaria	627	78.4	321	80.3	306	76.5	0.198
First health care provider for ARI related symptoms (n=696)							
VMW	241	34.6	71	19.7	170	50.7	<0.001
Public	103	14.8	89	24.7	14	4.2	
Private**	313	45.0	181	50.1	132	39.4	
Home care	39	5.6	20	5.5	19	5.7	
First health care provider for Diarrhoea (n=238)							
VMW	70	29.4	8	8.6	62	42.8	<0.001
Public	41	17.2	30	32.3	11	7.6	
Private**	110	46.2	48	51.6	62	42.8	
Home care	17	7.1	7	7.5	10	6.9	
First health care provider for Fever/Malaria (n=627)							
VMW	223	35.6	75	23.4	148	48.4	<0.001
Public	87	13.9	70	21.8	17	5.6	
Private**	262	41.8	148	46.1	114	37.3	
Home care	55	8.8	28	8.7	27	8.8	
Severity of symptoms							
Mild	199	24.9	112	28.0	87	21.8	0.120
Moderate	441	55.1	210	52.5	231	57.8	
Severe	160	20.0	78	19.5	82	20.5	
Number of symptoms							
One	29	3.6	29	7.3	39	9.8	0.308
Two	56	7.0	56	14.0	63	15.8	
Three or more	315	39.4	315	78.8	298	74.5	

*Within three months prior to the survey.

**Private clinic, pharmacy, traditional healer and other private practices.

VMWs from 36 villages (18 villages from each province) also participated in the study. Of them, 18 were providing M services, while the other 18 were providing M+C services. All of the VMWs were farmers, and 70% were male. The age of VMWs ranged from 20 to 66 (mean 42.4) years. The mean length of education VMWs had received was 5.8 years. The length of VMWs’ working experience providing services in the villages ranged from 32 to 100 (mean 60.7) months. Caregivers in M villages were in contact with VMWs having fewer months of working experience compared to caregivers in M+C villages (M 37.9 months *vs* M+C 83.5 months, p <0.001). The majority of caregivers (94.3%) knew the designated VMWs (M 90.0% *vs* M+C 98.5%, p <0.001) of their villages. Utilization rates for VMW services were higher in M+C villages compared to those in M villages (ever used VMWs services: M 69.0% *vs* M+C 80.8%, p <0.001; used VMWs’ child health services within the three months prior to the survey: M 22.8% *vs* M+C 51.8%, p <0.001).

Ages of children ranged from two to 59 (mean 27.8) months. The numbers of reported symptoms of ARI, diarrhoeal diseases and febrile illnesses occurring over the past three months prior to the survey were 696 (87.0%), 238 (29.8%), and 627 (78.4%), respectively. Private practices (private clinics, pharmacies, traditional healers and others) were the most common sources of care providers used by caregivers for the various childhood illnesses in M villages (ARI related symptoms: 50.1%, diarrhoea: 51.6%, fever: 46.1%), whereas VMWs were the most commonly used in M+C villages (ARI related symptoms: 50.7%, diarrhoea: 42.8%, fever: 48.4%). Compared to children from M villages, a higher proportion of children from M+C villages had experienced diarrhoea (23.3 *vs* 36.3%, p <0.001), while a lower proportion had experienced ARI related symptoms (90.3 *vs* 83.8%, p = 0.006).

### Sociodemographic characteristics and determinants of service utilization in M villages

Among 400 caregivers in M villages, 91 (22.8%) had utilized VMW services for their children’s illnesses within the three months prior to the survey (Table 2).

**Table 2:** Determinants of VMW service utilization by caregivers in M villages (n=400)

**Factors**	**VMW service**		**AOR**	**(95% CI)**	**p-value**
	**Users**	**Non-users**			
	**(n=91)**	**(n=309)**			
VMW’s education					
<5 years	40	168			
≥5 years	51	141	2.00	(1.05-3.83)	0.036
VMW’s experience					
<41.5 months	13	187			
≥41.5 months	78	122	11.80	(4.46-31.19)	<0.001
VMW’s service quality					
<4.1 points	27	229			
≥4.1 points	64	80	2.04	(1.01-4.11)	0.046
Caregiver’s age					
<28 years	55	162			
≥28 years	36	147	0.92	(0.50-1.68)	0.777
Number of children U5					
One	79	245			
Two or more	12	64	0.65	(0.30-1.45)	0.296
Distance from nearest public health centre					
<9.5 km	44	156			
≥9.5 km	47	153	3.03	(1.44-6.37)	0.004
Caregiver’s literacy					
Illiterate	34	125			
Literate	57	184	0.66	(0.351-1.24)	0.194
Caregiver’s occupation					
Farmer	86	277			
Other	5	32	0.89	(0.28-2.83)	0.846
Caregiver’s wealth index					
Low	19	77			
Middle	36	113	0.91	(0.45-1.85)	0.797
High	36	119	0.73	(0.36-1.49)	0.386
Child age					
<12 months	17	55			
12-59 months	74	254	1.04	(0.50-2.18)	0.918
Most recent symptom was Fever/Malaria					
Yes	81	240			
No	10	69	0.37	(0.16-0.87)	0.022
Severity of symptoms					
Mild	39	73			
Moderate	39	171	0.40	(0.21-0.76)	0.005
Severe	13	65	0.20	(0.08-0.47)	<0.001
Number of symptoms					
One	9	20			
Two	9	47	0.56	(0.16-2.01)	0.374
Three or more	73	242	0.72	(0.50-1.68)	0.530

The factors associated with VMW service utilization in M villages were as follows. Compared to caregivers whose VMWs had fewer years of education, caregivers whose VMWs had more years of education were twice as likely to have utilized VMW services (adjusted odds ratio [AOR] = 2.00; 95% confidence interval [CI] 1.05-3.83, p <0.036). Caregivers whose VMWs had longer (41.5 months or more) working experience as a VMW were more likely to have utilized VMW services compared to caregivers whose VMWs had shorter (less than 41.5 months) experience (AOR = 11.80; 95% CI 4.46-31.19, p <0.001). Compared to caregivers whose VMWs had lower service quality, those having VMWs with higher service quality were twice as likely to have utilized VMW services (AOR = 2.04; 95% CI 1.01-4.11; p = 0.046). Caregivers living farther (9.5 km or farther) from the nearest public health centre, meanwhile, were more likely to have utilized VMW services compared to caregivers living closer (less than 9.5 km) to the nearest public health centre (AOR = 3.03; 95% CI 1.44-6.37; p = 0.004).

On the other hand, compared with having a child who had malaria or fever most recently, having a child whose most recent symptoms fell into other categories were 63% less likely to have used VMW services (AOR = 0.37; 95% CI 0.16-0.87; p = 0.022). Further, compared to caregivers of children with mild symptoms, caregivers of children with moderate symptoms (AOR = 0.40; 95% CI 0.21-0.76; p = 0.005) and severe symptoms (AOR = 0.20; 95% CI 0.08-0.47; p < 0.001) were less likely to have utilized VMW services.

### Sociodemographic characteristics and determinants of service utilization in M+C villages

Among 400 caregivers in M+C villages, 207 (51.8%) had utilized VMW services for their children’s illnesses within the three months prior to the survey (Table 3).

**Table 3:** Determinants of VMW service utilization by caregivers in M+C villages (n=400)

**Factors**	**VMW service**		**AOR**	**(95% CI)**	**p-value**
	**Users (n=207)**	**Non-users (n=193)**			
VMW’s education					
<4 years	127	73			
≥4 years	80	120	1.48	(0.95-2.31)	0.085
VMW’s experience					
<72 months	85	119			
≥72 months	122	74	2.44	(1.52-3.94)	<0.001
VMW’s service quality					
<4.2 point	85	91			
≥4.2 point	122	102	0.84	(0.51-1.38)	0.487
Caregiver’s age					
<28 years	114	94			
≥28 years	93	99	0.82	(0.51-1.30)	0.397
Distance from nearest public health centre					
<25.5 km	127	73			
≥25.5 km	80	120	0.35	(0.21-0.56)	<0.001
Caregiver’s literacy					
Illiterate	117	118			
Literate	90	75	0.98	(0.58-1.65)	0.943
Caregiver’s occupation					
Farmer	198	172			
Other	9	21	0.43	(0.17-1.05)	0.062
Caregiver’s wealth index					
Low	69	46			
Middle	102	89	0.63	(0.37-1.07)	0.089
High	36	58	0.35	(0.18-0.68)	0.002
Child age					
<12 months	36	46			
12-59 months	171	147	1.29	(0.17-2.23)	0.358
Most recent symptom was Fever/Malaria					
Yes	158	148			
No	49	45	0.96	(0.56-1.62)	0.866
Severity of symptoms					
Mild	51	36			
Moderate	121	110	0.75	(0.43-1.31)	0.312
Severe	35	47	0.52	(0.25-1.05)	0.068
Number of symptoms					
One	21	18			
Two	30	33	1.01	(0.41-2.48)	0.979
Three or more	156	142	1.29	(0.61-2.74)	0.511

The factors associated with VMW service utilization in M+C villages were as follows. Caregivers whose VMWs had longer (72 months or more) experience as a VMW were more likely to have utilized VMW services compared to caregivers whose VMWs had shorter (less than 72 months) experience (AOR = 2.44; 95% CI 1.52-3.94; p <0.001).

On the other hand, living farther (25.5 km or more) from the nearest public health centre was negatively associated with caregivers’ utilization of VMWs’ services (AOR = 0.35; 95% CI 0.21-0.56; p <0.001). Compared to caregivers who had low wealth index scores, caregivers on the high end of the index were 65% less likely to have utilized VMW services (AOR = 0.35; 95% CI 0.18-0.68; p = 0.002). However, quality of VMW services was not significantly associated with service utilization in M+C villages (AOR = 0.84; 95% CI 0.51-1.38; p = 0.487), nor were any of the child-related factors.

### Caregivers’ Knowledge of childhood malaria management

Most of the caregivers demonstrated accurate malaria knowledge (Table 4). For example, they knew that mosquito bites cause malaria (98.4%), that bed nets should be used while sleeping to prevent malaria transmission (99.4%), that mosquito bites should be avoided (97.5%), that shivering (98.9%) and high fever (97.3%) are the main symptoms of malaria, that malaria is diagnosed by checking the blood (99.0%), and that anti-malarial drugs are the method of treatment (85.6%).

**Table 4:** Malaria knowledge of caregivers (Items of malaria knowledge index) (n=800)

**Items**		**n**	**%**
**Transmission route**	Mosquito bite (Yes)	787	98.4
	Coming close to malaria patient (No)	608	76.0
	Sharing food with malaria patient (No)	599	74.9
	Food poisoning/witchcraft (No)	592	74.0
	Cough and sneeze of malaria patient (No)	469	58.6
	Touching blood of malaria patient (No)	387	48.4
**Prevention**	Sleep under the bed net (Yes)	795	99.4
	Avoid mosquito bite (Yes)	780	97.5
	Wear long-sleeved shirts/pants (Yes)	757	94.6
	Clean bush around house (Yes)	731	91.4
	Cover water jars/tanks (Yes)	706	88.3
	Come back home before dawn (Yes)	664	83.0
	Use mosquito coil (Yes)	662	82.8
	Don't go close to malaria patient (No)	442	55.3
**Symptom**	Shivering (Yes)	791	98.9
	High fever (Yes)	778	97.3
	Headache (Yes)	735	91.9
	Sweating (Yes)	682	85.3
**Diagnosis**	Blood check (Yes)	792	99.0
	Only high fever (No)	286	35.8
	Only observation of provider (No)	188	23.5
**Treatment**	Anti-malarial drug (Yes)	685	85.6
	Antibiotic (No)	551	68.9
	Home remedy (No)	556	69.5

At the same time, some of them also believed in inaccurate information related to malaria management. Only about half of the caregivers understood that malaria cannot be transmitted through coughs or sneezes (58.6%), or by touching the blood of someone with malaria (48.4%). Similarly, only half understood that staying physically apart from malaria patients cannot prevent malaria (55.3%), and less than half correctly noted that malaria cannot be diagnosed from the mere presence of high fever (35.8%) or by a health care provider’s simple observation alone (23.5%). Moreover, more than a fourth of the caregivers believed that food poisoning or witchcraft could cause malaria (food poisoning or witchcraft cannot cause malaria: 74.0%).

### Determinants of knowledge on childhood malaria management

Caregivers of a child who had experienced fever or malaria symptoms most recently and who had sought care from VMWs as the first health care provider in M and M+C villages were included in the analysis to determine factors associated with knowledge of childhood malaria management (Table 5). Out of 223 eligible caregivers, 123 had high levels of knowledge and 100 had low levels of knowledge (“high” and “low” groups were created based on the median [3.92] knowledge index score).

**Table 5:** Factors associated with knowledge of childhood malaria management of caregivers (n=223)

**Factors**	Malaria knowledge*		**AOR**	**(95% CI)**	**p-value**
	**High**	**Low**			
	**(n=123)**	**(n=100)**			
VMW’s education					
<5 years	61	56			
≥5 years	62	44	2.00	(0.98-4.08)	0.057
VMW’s experience					
<72 months	78	56			
≥72 months	45	44	0.61	(0.29-1.32)	0.212
VMW’s service quality					
<4.2 points	27	53			
≥4.2 points	96	47	3.21	(1.34-7.66)	0.009
Caregiver’s age					
<27 years	68	47			
≥27 years	55	53	1.87	(0.84-4.17)	0.128
Household size					
<4 person	70	44			
≥4 person	53	56	0.93	(0.44-1.96)	0.840
Distance from nearest public health centre					
<10 km	78	35			
≥10 km	45	65	0.75	(0.33-1.72)	0.493
Caregiver’s literacy					
Illiterate	30	75			
Literate	93	25	9.91	(4.66-21.05)	<0.001
Caregiver’s occupation					
Farmer	117	95			
Other	6	5	0.38	(0.09-1.60)	0.186
Caregiver’s wealth index					
Low	26	37			
Middle	63	42	1.24	(0.55-2.80)	0.598
High	34	21	0.89	(0.34-2.32)	0.804
Child age					
<12 months	25	17			
12-59 months	98	83	0.78	(0.33-1.84)	0.564

* Malaria knowledge divided into two categories; High/Low, at the median (=3.92) of malaria knowledge index.

Caregivers whose VMWs had higher service quality exhibited higher knowledge levels on childhood malaria management compared to caregivers whose VMWs had lower service quality (AOR = 3.21, 95% CI 1.34-7.66; p = 0.009). Literate caregivers were also nearly ten times more likely to have higher knowledge levels compared to illiterate caregivers (AOR = 9.91, 95% CI 4.66-21.05; p < 0.001).

## Discussion

This study provides new insights into caregivers’ utilization of VMWs’ child health services in remote villages in Cambodia. First, the rates and determinants of VMW service utilization by caregivers were different between M villages and M+C villages. Second, the VMWs’ service quality and their length of experience working as a VMW were important determinants for caregivers’ utilization of child health services and their knowledge of childhood malaria control in VMW villages. Finally, some caregivers in M villages sought care for childhood illnesses from VMWs even though VMWs were technically providing only malaria control services.

The differences in VMW service utilization rates may be explained in part by the differences in circumstances under which villages were selected as VMW project sites and by discrepancies in the caregivers’ sociodemographic characteristics between M and M+C villages. Namely, M+C villages were selected as VMW project sites before 2004 because of their location (remote and closest to forests where malaria vectors breed) and high risk for malaria transmission. In contrast, M villages were selected after 2009 to expand VMWs’ malaria control services to villages at lower risk of malaria transmission but far (over 5 km) from public health centres [4]. The duration of VMW services being provided in M villages was much shorter compared to the situation in M+C villages. This could be one of the reasons that caregivers’ awareness of VMWs’ services was lower in M villages compared to M+C villages, as fewer caregivers had likely utilized VMW services in these settings. Additionally, one explanation for the much lower utilization rates observed in M than in M+C villages may be related to the limited range of services (only malaria control) available from VMWs in M villages as compared to M+C villages (malaria control services and childhood illness services).

The determinants of caregivers’ VMW service utilization were different between M and M+C villages. Namely, in M villages, factors linked to higher VMW service utilization rates were largely related to the VMWs characteristics, including higher education levels (caregivers twice as likely to use VMW services), longer experience working as a VMW (12 times more likely), and high service quality (twice as likely). Moreover, farther distance (9.5 km or more) between caregivers’ residences and the nearest public health centres was associated with a three times greater likelihood of utilizing VMW services for childhood fever or malaria with mild or moderate symptoms. This result could be related to the villages’ locations (M villages were less remote than M+C villages) and that only malaria control services were available from VMWs in M villages. Overall, caregivers in M villages selected to utilize VMW services when they judged their VMWs were capable and that their children’s conditions were appropriate to be treated by VMWs. An association between high quality of CHW services and high service utilization by caregivers has already been reported from remote and resource-poor settings including Nepal, Bangladesh, and Zambia [19,21,37-39].

On the other hand, in M+C villages, just three factors were significantly associated with caregivers’ service utilization. First, caregivers whose VMWs had more years of working experience were about two times more likely than those whose VMWs had fewer years experience to have utilized their VMWs’ services. Second, caregivers whose wealth index score was in the low or middle range were more likely than those on the high end of the wealth index to have utilized VMW services, possibly because such services were free and did not entail any extra transportation costs. Third, unlike the result from M villages and a previous study from Uganda [40], caregivers residing farther (25 km or more) from the nearest public health centre were less likely than caregivers residing closer to a public health centre to have utilized VMW services. Delays in medical supplies (VMWs in Kampong Thom province and Aya Hasegawa, personal communication, August 4, 5 and 6, 2012) could be one of the reasons for caregivers’ low VMW utilization rate in farther villages. The basic medication supplies for childhood illness management (antibiotics; cotrimoxazole, antipyretics; paracetamol, ORS and zinc) have been less stable than those for malaria control (ACT and RDTs) in remote villages. Overall, more than 50% of caregivers had utilized VMWs’ child health services within the three months prior to the survey in M+C villages. This could be because VMWs were sometimes the only health care provider available, in terms of affordability and accessibility, for caregivers in M+C villages. Under these circumstances, caregivers may be electing to utilize VMW services simply because such services are there.

The quality of VMWs’ services was positively associated with caregivers’ knowledge of childhood malaria management in VMW villages. Among caregivers whose children had fallen ill with fever or malaria, knowledge levels regarding childhood malaria management were about three times higher if the service provided by the local VMWs was of high quality compared to those who had VMWs with low service quality. The majority of caregivers had accurate knowledge of childhood malaria management but, at the same time, many also believed in spurious rumours or traditional sayings, for example, that “food poisoning or witchcraft” is a transmission route for malaria, or that not “going close to a malaria patient” could prevent malaria transmission. Caregivers’ inaccurate perceptions or beliefs can sometimes cause delays in seeking care for childhood illnesses, which can lead to symptoms becoming more severe and more complicated [41,42]. Therefore, there is a clear need for VMWs to update caregivers’ knowledge on childhood illness management so they can protect their children. Also, VMWs need to pass on accurate and up-to-date information to caregivers. The provision of close monitoring with prompt feedback, on-site supervision, and regular refresher training [25,33,39] by CNM staff members, in collaboration with local public health centre staff members, are necessary to improve the service quality of VMWs.

Among all study participants, about a third never attended school and roughly half were illiterate – rates higher than the corresponding national averages [7]. Moreover, literate caregivers were about ten times more likely to exhibit high knowledge levels regarding childhood malaria management than were illiterate caregivers. It is therefore important to improve childhood malaria management knowledge of caregivers. VMWs, as community members and the most commonly accessed health care providers in remote villages, are well suited to provide such knowledge to caregivers with user-friendly and culturally acceptable methods. VMWs’ use of their own regional dialect and pictorial explanations, rather than reliance on printed information materials, could help substantially improve the knowledge of caregivers.

Notably, even though VMWs in M villages provided only malaria control services, caregivers in these villages also sometimes visited VMWs to seek care for their children’s ARI or diarrhoeal diseases. Indeed, only providing malaria control services may not be enough, because about 90% of children had ARI related symptoms and 30% of children had diarrhoea in the study villages. Moreover, more than 90% of the children surveyed had experienced two or more symptoms at once. Previous studies have reported possible risks associated with providing treatment for a single disease in isolation. For example, the risk of providing malaria treatment for ARI cases led to delays in ARI treatment and thus created severe ARI cares in Zambia [43]. Similarly, difficulties in dealing with recurring childhood illnesses by CHWs trained to treat only a single disease were highlighted in studies from Tanzania, Nicaragua, and Bangladesh [27,44-46]. Hence, the concept of “integration” is important and may prove more effective than single-disease interventions [21-23,47]. The VMW project could be successfully scaled up in terms of the number of VMWs and the range of health services provided without degrading the quality of their malaria control services [26]. Against this backdrop, scaling up VMW capacities from malaria control services (M) to malaria control and child health services (M+C) can be an effective strategy to simplify care-seeking procedures for caregivers, providing a one-stop integrated intervention approach and minimizing the time to first diagnosis and treatment for sick children.

Findings from this study should be considered in the context of some limitations. First, because the field survey was conducted during Cambodia’s heavy rainy season, accessibility of the villages had to be taken into consideration when selecting study sites. However, the farthest villages from public health centres were selected among the subset of accessible villages. Second, caregivers were asked about childhood illnesses occurring within the three months prior to the survey. This may have introduced recall bias. To minimize the recall bias, however, data were collected by experienced and trained interviewers with on-site supervision. Third, all the data used in this study were self-reported by caregivers and VMWs. There was thus a possibility of courtesy bias. However, self-reported data were double-checked with VMWs’ monthly records [33]. Also, the experienced and trained interviewers provided a comfortable environment for participants to answer the questions.

Despite these limitations, this is the first study to identify the determinants of caregivers’ utilization of VMWs health services following the project’s scale-up and integration of child health services with VMWs’ existing malaria control roles in M+C villages. The findings provide further insights into the community needs related to child health services in remote and resource-poor settings. In addition, this study broke new ground in examining the association between VMWs’ service quality and caregivers’ service utilization and knowledge, targeting both service providers and service users. By combining and analysing data from providers’ and users’ sides alike, this study provided a more accurate evaluation of the intervention in context.

## Conclusions

This study has demonstrated that the service quality of VMWs and their length of experience working as a VMW are important determinants for caregivers’ utilization of child health services and their knowledge of childhood malaria control in VMW villages. VMWs are one of the most common child health care providers in VMW villages. Furthermore, caregivers are seeking VMWs for childhood illnesses even if they are providing only malaria control services. Therefore, there is a potential need for scaling up VMWs’ capacity by integrating child health services into their existing mandate to provide malaria control services, in order to further improve child health status in remote villages of Cambodia.

## Abbreviations

ACT: Artemisinin-combination therapy; AOR: Adjusted odds ratio; ARI: Acute respiratory infections; ASEAN: Association of Southeast Asian Nations; CDHS: Cambodia Demographic and Health Survey; CHW: Community health worker; CI: 95% confidence interval; CNM: Cambodian Government’s National Centre for Parasitology, Entomology and Malaria Control; GFATM: Global Fund for AIDS, TB and Malaria; M: Designates villages incorporating malaria control interventions only; M+C: Designates villages incorporating malaria control interventions and childhood illness management interventions; ORS: Oral rehydration salts; PASW: Predictive analytics software; RDTs: Rapid diagnosis tests; SD: Standard deviation; SPSS: Statistic package for social science; VMW: Village malaria worker; WHO: World Health Organization.

## Competing interests

The authors declare that they have no competing interests.

## Authors’ contributions

AH conceived the study, contributed to the study design, conducted the fieldwork, analysed the data, and wrote the manuscript. JY contributed to the study design, statistical analysis, interpretation of the data and improved the manuscript. PL and CN supervised fieldwork and improved the manuscript. MJ monitored the study progress and provided guidance to improve the manuscript. All authors read and approved the final draft.
